# Innate immunity and microbial dysbiosis in hidradenitis suppurativa – vicious cycle of chronic inflammation

**DOI:** 10.3389/fimmu.2022.960488

**Published:** 2022-07-28

**Authors:** Divya Chopra, Rachel A. Arens, Watcharee Amornpairoj, Michelle A. Lowes, Marjana Tomic-Canic, Natasa Strbo, Hadar Lev-Tov, Irena Pastar

**Affiliations:** ^1^ Wound Healing and Regenerative Medicine Research Program, Dr. Phillip Frost Department of Dermatology and Cutaneous Surgery, University of Miami Miller School of Medicine, Miami, FL, United States; ^2^ College of Medicine, University of Toledo College of Medicine and Life Sciences, Toledo, OH, United States; ^3^ Laboratory for Investigative Dermatology, The Rockefeller University, New York, NY, United States; ^4^ Department of Microbiology and Immunology, University of Miami Miller School of Medicine, Miami, FL, United States

**Keywords:** hidradenitis suppurativa, acne inversa, innate immunity, skin – immunology, antimicrobial peptides (AMPs), complement - immunological terms, microbiome and dysbiosis, biofilm

## Abstract

Hidradenitis Suppurativa (HS) is a chronic multifactorial inflammatory skin disease with incompletely understood mechanisms of disease pathology. HS is characterized by aberrant activation of the innate immune system, resulting in activation of pathways that aim to protect against pathogenic microorganisms, and also contribute to failure to resolve inflammation. Imbalance in innate immunity is evident in deregulation of host antimicrobial peptides (AMPs) and the complement system associated with the microbiome dysbiosis. The pathology is further complicated by ability of pathogens associated with HS to overcome host immune response. Potential roles of major AMPs, cathelicidin, defensins, dermcidin, S100 proteins, RNAse 7 and complement proteins are discussed. Dysregulated expression pattern of innate immunity components in conjunction with bacterial component of the disease warrants consideration of novel treatment approaches targeting both host immunity and pathogenic microbiome in HS.

## Introduction

Hidradenitis suppurativa (HS), also known as acne inversa, is a chronic, highly burdensome, inflammatory cutaneous disease that is associated with systemic manifestations. HS has a global prevalence ranging between 0.00033% to 4.10% (1). The onset of HS usually occurs in young adulthood to middle adulthood (1–3). Patients generally develop one or a few painful nodules or abscesses in intertriginous areas, such as armpits, groin and perianal area, in the early stages. The disease often progresses to form tunnels (also called sinus tracts or fistulas) and scars in more advanced stages. HS is recognized as a multifactorial disease; current evidence points to genetic predisposition, hormonal imbalance, lifestyle factors, and some unique features of the affected skin areas contribute to disease development (1, 2, 4).

Aberrant activation of the innate immune system is another major trait of HS. The intertriginous areas affected by HS are prone to increased mechanical friction causing cutaneous microinjuries in skinfolds which can lead to stimulation of innate immune responses and allow for the invasion of microorganisms to hair follicle and dermis (1, 2, 5). HS lesions commonly cause disfigurement, itch, malodorous pus, and movement restrictions, impacting patients’ quality of life (1–3, 6). The therapeutic options of HS are currently limited, including medical and surgical therapy, and are supported by limited data. Only a single drug has completed the US Food and Drug Administration approval process based on two large randomized controlled trials. Therefore, HS remains challenging to treat due to the complexity of the disease pathology, limited therapies and an enormous impact of the quality of life of patients (2, 5, 7).

Antimicrobial peptides (AMPs) are major component of the cutaneous innate immunity produced by epithelial and immune cells and possess direct bactericidal activity (7). Multiple AMPs are made in the skin, providing the cutaneous barrier with broad spectrum antimicrobial activity making them an essential aspect of the innate immune system. Some AMPs are constitutively expressed to keep the skin microbiota in balance while others are induced by the presence of pathogens or wounding and are also found deregulated in pathologic conditions including HS (8–12). Furthermore, the complement system, an immune effector system involved in the host response to microbes, is also dysregulated in HS (13, 14). Here we review the role of innate immunity in HS with focus on antimicrobial peptides and the complement system and their role in the pathogenesis of the disease. The interplay between microbial dysbiosis and deregulation of components of the innate immune system in HS pathology is discussed, with the focus on understanding the mechanistic contributions of both host response and microbiota to disease pathology.

## Microbial dysbiosis in HS

The balance between commensal microbiota contributes to, and is a hallmark of cutaneous homeostasis (15). In contrast, microbial dysbiosis is a well-established feature of HS (16, 17). Compared to other skin loci, intertriginous areas affected in HS have a high density of pilosebaceous-apocrine units, higher temperature and moisture, but lower oxygen availability all contributing to specific microbiome composition and increased risk of dysbiosis (1, 2, 5, 18). Systemic factors such as obesity, diabetes and nicotine use can also contribute to microbial dysbiosis and HS pathogenesis (1, 19, 20), and may correlate with the altered gut microbiome found in HS patients (21, 22). Although it remains to be resolved if dysbiosis is a primary trigger or a consequence of HS, multiple studies provide insights into the microbiome composition at early and advanced stages of the disease. The predominance of commensal coagulase-negative *staphylococci* (CoNS) and *Propionibacterium* has been associated with early HS lesions (23, 24). However, in advanced stages of HS, CoNS and additional commensals including *Cutibacterium* and *P. acnes*, are significantly diminished, while pathogenic *Staphylococcus aureus* persists (24–26). Gram-negative anaerobic pathogens *Porphyromonas spp*, *Peptoniphilus spp* and *Prevotella spp* become predominant in HS tunnels and advanced stages of the disease (24, 27, 28). These pathogens are proposed to contribute to biofilm formation found inlesional skin and tunnels (29–31).

Chronic HS lesions and tunnels contain keratin debris and hair fragments that may serve as surfaces for pathogenic anaerobes to anchor and initiate biofilm growth (29). Polymicrobial biofilms containing *Porphyromonas* and *S. aureus* have been associated with pathogenesis of periodontal disease (32); however interspecies interactions and their crosstalk with the host innate immunity in HS remain to be elucidated. Biofilms may not only cause an inappropriate host response contributing to perpetual inflammation but are also challenging to treat due to antibiotic and antimicrobial resistance (31, 33). Bacterial biofilms can also evade components of the innate immune system, including complement and AMPs (34–36).

## The role of AMPs in HS pathology

AMPs are a key part of the innate immune system of the skin with a critical role in preventing overgrowth of commensal microbiota, preventing invasion by pathogens, and initiating an inflammatory cascade aimed at wound healing (37). AMPs are especially important in the intertriginous regions as the environment is prime for microorganism growth. Without the appropriate expression of AMPs, the intertriginous skin may be overwhelmed by local commensals and is left vulnerable to colonization by pathogens. Importantly, dysregulation of AMPs that is observed in HS can contribute to the chronicity of inflammation, and microbial dysbiosis. Herein, we outline the role of the major AMPs, cathelicidin, defensins, dermcidin, S100 proteins and RNases in cutaneous homeostasis and their associated roles in HS pathology (**Table 1**).

**Table 1:** Overview of major AMPs associated with HS pathology.

AMP	Charge	Antimicrobial activity	Expression in normal skin	Expression in HS	Methods of detection in HS	Chemokinetic activity	Ref.
LL-37	Cationic	Broad spectrum	Constitutive low levels in keratinocytes and mast cells	Induced in follicular keratinocytes and neutrophils	qPCR, IHC, ELISA, microarray	Neutrophils, dendritic, mast and T cells	(10, 37–45)
Defensins	Cationic	hBD-1 and -2 mainly Gram-negative bacteria, hBD-3 broad spectrum	hBD-1 constitutive low expression; hBD-2 and hBD-3 low expression in keratinocytes	hBD-1 suppressed/not regulated in keratinocytes; hBD-2 induced in keratinocytes and dermal macrophages; hBD-3 induced/not regulated in keratinocytes	qPCR, IHC, ELISA	Dendritic and T cells, promotes histamine release from mast cells	(44, 46–63)
DCD	Anionic	Mainly Gram-negative bacteria	Constitutive expression in eccrine sweat glands	Suppressed in eccrine sweat glands	qPCR, IHC, ELISA	N/A	(9, 59, 60, 64–68)
S100	Cationic	Broad spectrum	Rarely detected in healthy skin	Induced in epidermal keratinocytes	qPCR, IHC, ELISA, proteomics	Neutrophils and macrophages	(46, 51, 52, 54, 59, 60, 69–76)
Rnase7	Cationic	Broad spectrum	Constitutive expression in keratinocytes	Repressed/Induced/not regulated in keratinocytes	qPCR, IHC, ELISA	N/A	(44, 46, 59, 77)

The main AMPs with altered expression in HS include LL-37, hBD-1, hBD-2, hBD-3, DCD, S100s proteins, and RNAse 7. Multiple different methods have been utilized to assess their expression, including quantitative PCR (qPCR), immunohistochemistry (IHC), enzyme-linked immunoassay (ELISA) and proteomics. Some AMPs are consistently found deregulated including LL-37, hBD-2, DCD, and S100s proteins. However, the deregulation and directionality were not consistent for hBD-1, hBD-3, and RNAse 7 across studies. Ref.=references.

### Cathelicidin role and function in skin and HS

A single cathelicidin gene in humans, *cathelicidin antimicrobial peptide* (*CAMP*) encodes for a precursor protein, human cationic antimicrobial protein, hCAP18, which is proteolytically processed by serine proteases into multiple different peptides, including LL-37 (10). Cathelicidins have broad spectrum antimicrobial activity and are constitutively expressed by follicular keratinocytes and mast cells in healthy skin. The production of LL-37 is induced by cutaneous inflammation, often at the hands of pathogens (10, 38, 39). LL-37 is also present in sweat and wound fluid (38, 78). Specifically, LL-37 is induced in acute wounds with a diminishing concentration as the inflammation resolves and wound progresses to healing (79). Several factors determine expression of LL-37. *CAMP* is under transcriptional control by Vitamin D response element, and *CAMP* expression is strongly induced by Vitamin D in all skin cell types (10, 40). Vitamin D deficiency has been associated with multiple inflammatory skin diseases including psoriasis, rosacea, atopic dermatitis, and systemic lupus erythematous (80–82), while association with HS has been reported (83) but remains to be thoroughly investigated. The enzymatic processing of hCAP18 in the skin is controlled by serine proteases kallikrein 5 and kallikrein 7 (KLK5, KLK7), which are also induced by Vitamin D and pH changes (41, 84, 85). KLK and its substrates are found in the dark cells in eccrine sweat glands but not sebaceous glands, hair follicles, keratinocytes, or elsewhere in the pilosebaceous unit (86).

Multiple studies have found LL-37 expression to be increased in the skin of HS patients on both mRNA and protein levels (42–44) (**Table 1**). In addition to its antimicrobial activity, LL-37 has prominent immunomodulatory activity, including chemotaxis of neutrophils, monocytes, mast cells, and T cells, and can influence Th1/Th17 cell maturation (37, 45). Neutrophils drawn to the inflammation further promote the process by releasing additional cathelicidin from granules (37). Chemotactic function of LL37 attracts CD4 T cells and dendritic cells, which further release TNF-α, IL-6, and Il-12. The upregulation of these inflammatory markers results in the Th1/Th17 phenotype, independent of antigen-presenting cells (43), while LL-37 may also act as T-cell autoantigen in psoriasis (87). Additional studies have found the progression of HS severity to be correlated with increased levels of these pro-inflammatory cytokines promoted by LL-37, including IL-17 and TNF-α (46, 88–90), suggesting the potential therapeutic targeting of LL-37. LL-37 also promotes proliferation of keratinocytes by an anti-apoptotic mechanism (91), which may be contributing to epidermal hyperplasia and proliferation of epithelial strands further stimulating formation of tunnels. Bacterial aggregates in these tunnels may form biofilms stringently attaching to epithelial tunnels and amplifying inflammation both locally and systemically (4, 12), though various mechanisms including increase of lipocalin-2 (6, 92, 93). Overall consistent upregulation of LL-37 in HS may allow for the local inflammation to progress to a systemic disease creating a positive feedback loop due to deregulated inflammation and microbial dysbiosis.

Recent findings revealed a contribution of LL-37 to bacterial antibiotic tolerance in S. aureus. LL-37 induced *S. aureus* tolerance to daptomycin by activating staphylococcal GraRS two-component system, leading to increased peptidoglycan formation, a key component of biofilm formation (94). LL-37 has also been identified to reduce *S. aureus* susceptibility to vancomycin (95). These findings underscore complexity of host-pathogen interaction in inflammatory conditions associated with AMP upregulation and warrants further investigation in HS considering the prevalence of *S. aureus* (96, 97).

### The role of defensins in cutaneous immunity and deregulation in HS

Defensins are cysteine-rich cationic peptides grouped into α or β; α-defensins are mainly expressed in leukocytes and Paneth cells while β-defensins are found in various epithelial cells, including keratinocytes (47, 48). There are multiple β-defensins; most important are human β-defensin-1 (hBD-1), hBD-2 and hBD-3, all of which are expressed by keratinocytes in response to inflammation or in response to pathogens (49). Unlike cathelicidins, defensins are not proteolytically processed to generate different defensin-peptides, rather they are encoded by multiple defensin genes. hBD-1, -2, and -3 are encoded for by *DEFB1*, *DEFB4* and *DEFB103*, respectively. *DEFB2*, like *CAMP*, is also under transcriptional regulation of vitamin D (50) and *DEFB4* is significantly upregulated in HS lesional skin (44, 46, 51, 52).

hBD-1 and hBD-2 are mainly effective against Gram-negative organisms while hBD-3 has broad spectrum activity (49, 53). Fewer studies have focused on hBD-1; however, it has been consistently found to be decreased in HS lesions (**Table 1**) (54, 55). As hBD-1 is constitutively expressed in healthy skin (56), it forms an important part of the cutaneous innate immune system. Without sufficient hBD-1 patients may be susceptible to commensal overgrowth in early stages of the disease, allowing for microbial dysbiosis as the disease progresses.

hBD-2 and hBD-3 are not constitutively expressed but upregulated at a transcriptional level in response to microorganisms and also increased levels of TNF-α, a hallmark proinflammatory cytokine of HS (**Table 1**) (13, 49, 53). Increased lesional hBD-2 was strongly correlated with higher levels of IL-20 and IL-22 (54). hBD-2 has well-described chemotactic abilities, as it attracts dendritic cells, memory T cells, and promotes histamine release from mast cells (57, 58), therefore primarily contributing to prolonged inflammation unable to clear bacterial biofilms in HS tunnels. In addition, both mRNA and protein levels of hBD-2 were released by dermal macrophages at a significantly higher level in HS lesions, further amplifying pro-inflammatory signature of HS (51). In contrast to upregulation of hBD-2, conflicting data were reported on hBD-3 indicating either upregulation (42, 46, 54, 55, 59) or lack of regulation compared to non-lesional skin (44, 60). Both hBD-2 and hBD-3 can stimulate keratinocyte proliferation (98). As the levels of these AMPs increase in HS lesions, keratinocytes multiply within the pilosebaceous unit, leading to hyperkeratosis and plugging of the hair follicle which may serve as fuel for microbial dysbiosis and biofilm formation. Overall, hBDs may primarily contribute to HS pathogenesis by promoting keratinocyte proliferation and inflammatory cell chemotaxis.

### Dermcidin function in skin and suppression in HS

Like cathelicidin, dermcidin originates from a single gene, *dermcidin (DCD)*, which encodes for an AMP with broad-spectrum activity. DCD is proteolytically processed by cathepsin D (CatD) into DCD-1 and DCD-1L, and others (9, 99, 100). DCD is constitutively and specifically expressed by eccrine sweat glands, especially on the face and hands, but could not be isolated from apocrine sweat glands (9, 64). Unlike most other AMPs, DCD is anionic and has been shown to form large pores in the membranes of Gram-negative bacteria (65, 66). Interestingly, DCD-1 is amphipathic, forming a large complex alongside the bacterial membrane, which is stabilized by zinc ions in sweat, allowing ion channel formation on the pathogens’ membrane (8). DCD is not expressed by keratinocytes in the presence of inflammation like the other AMPs, leaving the current understanding of its function as maintenance of commensal skin microbiota and prevention of pathogens invasion largely unknown (67).

DCD was significantly downregulated in HS lesional skin on mRNA and protein level (**Table 1**) (60, 101), suggesting that insufficient levels of DCD may allow overgrowth of Gram-negative bacteria in HS tunnels. However, like the other AMPs, DCD-derived peptides can promote epidermal inflammation *via* TNF-α, IL-8, CXCL 10 and CCL20 (102), limiting the opportunity for therapeutic targeting of DCD in HS. Further investigation of the role of DCD in HS is needed to shed a light on this AMP and its potential contribution to microbial dysbiosis at different stages of disease.

### The role of S100 proteins in HS

The S100 protein family, also known as alarmins, consist of at least 21 calcium-binding cytosolic proteins which can be divided into 3 divisions: those with intracellular function only, those with extracellular function only, and those with both. By altering calcium signaling, S100 proteins can serve as intracellular regulators and extracellular signaling molecules in either a paracrine or autocrine manner (103). The S100 protein family has a wide range of function including regulating cell migration, proliferation, differentiation, and apoptosis as well as modulating inflammation and energy metabolism (104, 105), and are made by keratinocytes, dendritic cells, neutrophils, and macrophages (69, 106).

In healthy skin, S100A7, S100A8 and S100A9 are rarely detected at the protein level, while increased levels are well documented in many inflammatory skin conditions such as psoriasis, atopic dermatitis, and mycosis fungoides. Among the S100 protein family, S100A7 (psoriasin), S100A8 (calgranulin A), S100A9 (calgranulin B), S100A12 (calgranulin C) and S100A15 (koebnerisin) have well documented antimicrobial activity (107). S100A7 and S100A15 have strong bactericidal activity against *Escherichia coli* with weaker preference for Gram-positive bacteria while S100A8/S100A9 and S100A12 have preferential activity against fungi and viruses (70, 107, 108). S100A8 and S100A9 also form a heterodimer known as S100A8/S100A9 or calprotectin which plays an integral role in acute and chronic inflammation (11).

Multiple studies have confirmed increased levels of S100 proteins in HS (**Table 1**). Increased expression of S100A7 at both the mRNA and protein levels was found in lesional HS skin (46, 51, 52, 60, 71). S100A8, S100A9, S100A12 and S100A15 have also been shown to be increased in lesional skin in HS (13, 46, 52, 54, 60, 109). Overexpression of S100A15 and S100A12 was noted in perilesional skin as well, which suggests a possible role in early pathogenesis of HS by stimulating keratinocyte proliferation in the perifollicular region (13, 109). The increase of S100 proteins may occur in response to elevated inflammatory cytokines such as interleukin (IL)-17 and IL-22, as in psoriasis (110, 111). The overall increased levels of S100 proteins in HS can further elevate level of inflammation due to known chemotactic function of S100A7 and S100A8/S100A9 complex, subsequently increasing expression of proinflammatory cytokines and inducing a positive feedback loop, while microbial dysbiosis persists. The family of S100 proteins exerts its antimicrobial function through sequestration of Mn^+^ and Zn^+^ ions required for bacterial metabolism (112); however, excessive sequestration can also negatively affect host response e.g. impact Zn^+^-dependent DCD function (8) and further diminish antimicrobial response.

### Dysregulation of RNAse 7 in HS

RNAse 7 is one of eight enzymes in the RNAse A superfamily. The members of this family have been demonstrated to have a wide range of action including diverse immunomodulatory, angiogenic, and neurotoxic effects (113). A few members of this family also exert antimicrobial action including RNAse 3, 4, 7, 8, and 9 (114). RNAse 7 has a broad-spectrum antimicrobial activity against Gram -positive and -negative bacteria (113). RNAse 7 is constitutively expressed in keratinocytes and exists in high quantities in all layers of the epidermis with a particular concentration in the stratum corneum and in follicular epithelium (77, 115, 116), indicating its role in HS.

Currently, the relationship of RNAse 7 in HS skin has not been clearly defined and was primarily evaluated at mRNA levels (**Table 1**). RNAse 7 mRNA has been shown to be increased (46),. or decreased (59) in HS lesional skin when compared to heathy skin; not different between lesional and non-lesional HS skin (44), and increased in lesional HS skin compared to perilesional skin (117). There was significantly increased RNAse 7 mRNA in HS lesional skin compared to chronic venous leg ulcers (44). Overall induced expression of RNAse 7 can be attributed to higher levels IL-1β, IL-17, and IFN-γ in HS known to induce expression of this his AMP (77, 113). RNAse 7 can also promote rapid sensing of bacterial and human DNA by plasmacytoid dendritic cells resulting in enhanced production of TNF-α and IFN-α (118) contributing to perpetual cycle of inflammation in HS.

## Complement system and its role in disease pathogenesis

The complement system is a part of innate immunity that acts as first line defense against pathogens and altered host cells by augmenting opsonization of pathogens and promoting inflammatory processes. This system is largely composed of plasma proteins synthesized in the liver but also includes inactive precursor proteins on cell membranes (119). Because serum is restricted in areas were complement activation is needed, these proteins can also be made by a variety of cells including epithelial cells, endothelial cells, and immune cells (120, 121).

Activation of the complement system can be achieved by three distinct pathways – classical (CP), lectin (LP), and alternative (AP), each leading to a common terminal (cytolytic) pathway initiated by different recognition molecules (122, 123). All three pathways ultimately form C3 convertases to cleave C3 into C3a and C3b. C3a is an anaphylatoxin with a largely proinflammatory response including induction of oxidative burst in macrophages and neutrophils, histamine production *via* basophils to induce vasodilation, and increased vascular permeability (124). C3b acts as an opsonin which will target apoptotic cells, pathogens, and immune complexes for phagocytosis. Once levels of C3b have reached a threshold of activation, C3b can bind to a C3 convertase, either C4bC2a or C3bBb, to form C5 convertase which will cleave C5 to produce C5a. C5a in combination with the membrane attack complex (MAC) which consists of a polymer of C5b, C6, C7, C8, and C9 initiates a terminal pathway to bacterial cell lysis (120, 122, 125).

As a very dangerous cytolytic system, complement activation in the CP and LP pathways are tightly regulated in physiological conditions by many plasma proteins to ensure that only infection will stimulate a full response (122). Both inefficient activation and over stimulation of complement have been found to be associated with increased susceptibility to infections, while the imbalance of complement activation contributes to development of chronic inflammation (122, 126).

Complement is essential in maintaining cutaneous health by maintaining healthy skin microbiome and modulating inflammatory responses (127–129). Human keratinocytes produce several complement proteins including C3, C4, and complement factor B (FB) which is induced by IL- 1α, IFN-γ, and TNF-α (121). Keratinocytes also play a significant role in regulating response of complement proteins. Keratinocytes can synthesize soluble complement inhibitors like factor H and factor I, complement receptors CR1, cC1qR, C5aR1, and CR2 and cell-bound complement regulator proteins membrane cofactor protein (MCP/CD46), decay-accelerating factor (DAF), and protectin (CD59) (14). FH and FI production can be stimulated locally by IFN-γ which can then protect the epidermis from damage caused by C3, C4, and FB (130, 131).

**Figure 1:**
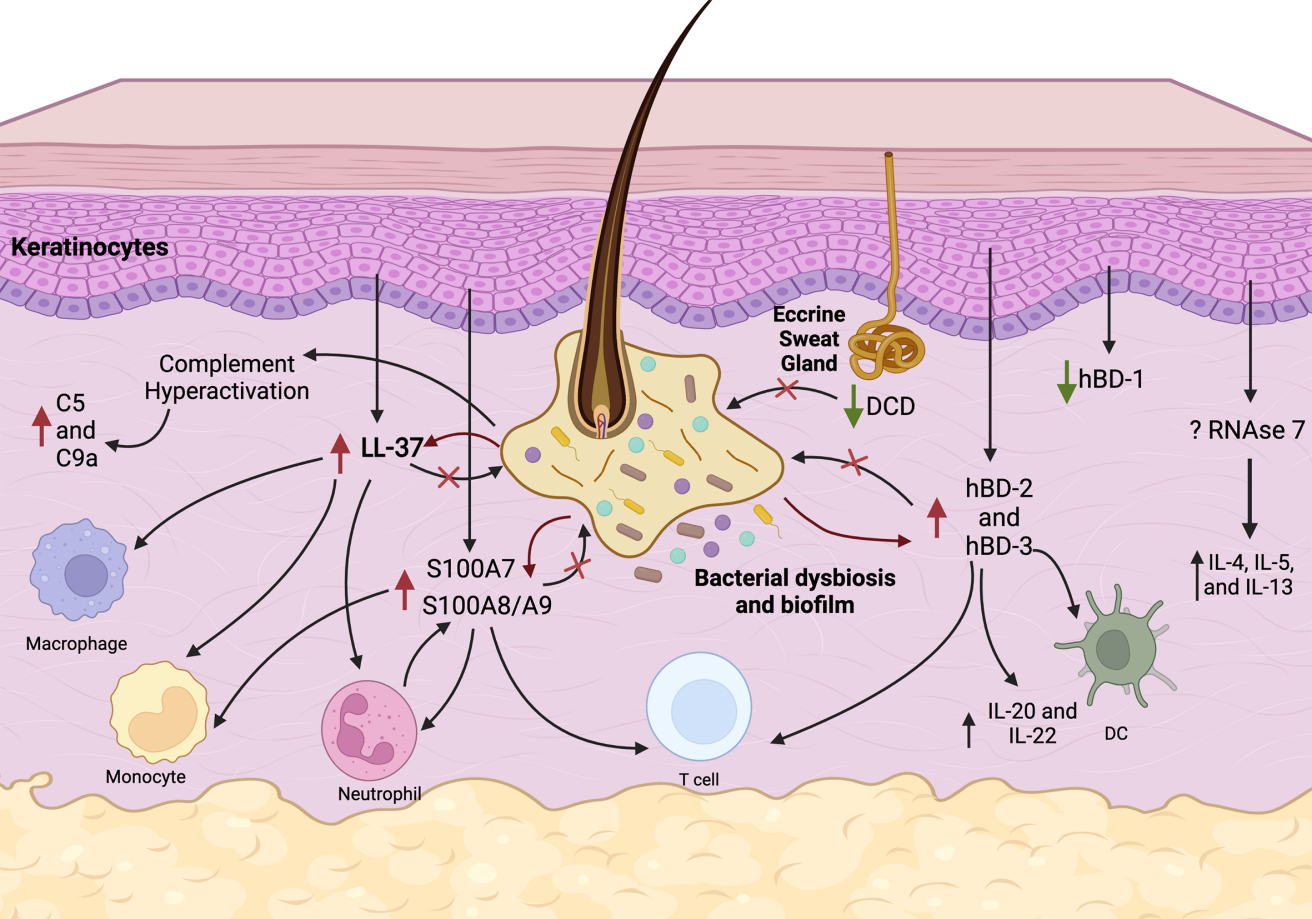
Proposed pathways of AMPs and complement deregulation in HS. Follicular inflammation, microbial dysbiosis and biofilm stimulate keratinocytes to overexpress LL-37 (cathelicidin), S100A7, S100A8/A9, hBD-2, and hBD-3. Immunomodulatory function of these AMPs includes chemotaxis of macrophages, monocytes, neutrophils, T cells, and dendritic cells (DC) and production of cytokines. Complement pathways are hyperactivated in HS causing elevated C5a and C9 levels. Constitutive production of hBD-1 from keratinocytes and DCD production from eccrine sweat glands is inhibited in HS skin which may result in microbial dysbiosis. Overall, these events lead to a persistent vicious cycle of chronic inflammation, which is ineffective in eliminating pathogens and biofilms.

Complement activation in HS is thought to be primarily systemic response (132). All three pathways, CP, LP, and AP, are thought to contribute the complement activation in HS (133). Ghias et al. suggested that the initial stimulus was follicular rupture releasing microbes and keratin which would trigger production of C3a, C3b, and C5a (127). This would in turn lead to bacterial opsonization, keratin phagocytosis, neutrophil chemotaxis, mast cell degranulation, and cell lysis through the MAC complex. Additionally, complement activation triggers inflammasomes to produce IL-1ß. IL-1ß further induces a cascade of proinflammatory cytokine release, AMP production, and DC activation (46, 127). A transcriptome study of the HS skin showed C1q, C2, and factor B genes were upregulated, whereas factor H, factor D, and C7 were downregulated. In the serum proteome, C5a was found upregulated, and C4b, C3, C3b, and iC3b were downregulated (133). Levels of C5a and the components of the MAC, C5b-C9, are significantly elevated in HS patients when compared to healthy subjects. When stratified amongst Hurley stages, levels of C5a and C5b-C9 were highest in patients with mild disease than those with more severe disease. However, results from a recent study reported no evidence of elevated plasma levels of C5b-9 in patients with HS despite decreased C3/C3d ratio (134). These results suggest that complement hyperactivation which is likely a consequence of initial dysbiosis of microbiota (**Figure 1**), plays an important role in early disease development in HS rather than being a sequela of chronic inflammation in more progressed disease (132), although it is also possible that there is increased activity of complement including opsonization due to increased C3b levels but without increased systemic terminal complement pathway (135).

Importantly, C5a is a critical stimulator of TNF-α release. In studies, purified blood mononuclear cells (PBMCs) from HS patients did not produce proinflammatory cytokines upon stimulation with bacterial ligands (136) but did overexpress TNF-α when in the presence of patients’ plasma. These findings support the idea of HS stemming from dysregulated innate immunity in conjunction with microbial dysbiosis. TNF-α in turn stimulates upregulation of C3 (137), FB in the alternative pathway (138), and surface regulatory proteins like CD59 (139). TNF-α overexpression was attenuated after introduction of an anti-C5a monoclonal antibody, suggesting an important relationship between C5a and TNF-α in worsening inflammatory responses in HS (132) and supporting therapeutic targeting of C5Aa (127).

Pathogens have developed complement evasion strategies to escape host innate immunity. Methods of complement evasion are highly conserved across the classes of pathogens including bacteria, virus, and fungi. Common strategies are inactivation *via* protease secretion, complement disguise using regulatory molecules, and expression of direct inhibitors (140). *Streptococcus pyogenes* and *S. aureus* have adapted to develop methods for complement evasion. *S. pyogenes* utilizes the M protein, a cell membrane-bound polymorphic protein, to defend against complement-mediated phagocytosis by binding regulatory proteins such as FH and C4BP to the bacteria surface. Additionally, MAC-mediated bacterial lysis is inhibited by *S. pyogenes* due to the thick outer peptidoglycan layer (141). Furthermore, the MAC complex is rendered useless directly by streptococcal inhibitor of complement (SIC) expression (142) and indirectly by expression of vitronectin-binding proteins (143). Similarly, *S aureus* evades complement through use of regulatory proteins such as FH and inactivation *via* a staphylococcal complement inhibitor (SCIN). *S aureus* secretes extracellular protein B (Ecb) and staphylococcal binder of IgG (Sbi) to enhance deposition of FH onto staphylococcal membrane (144, 145). SCIN targets C3BbBb to inhibit formation of C3a, C3b, and C5a and thus preventing bacterial opsonization and decreasing phagocytic cell chemotaxis. *S. aureus* has also developed mechanisms capable of evading killing by additional host MAC-domain protein, Perforin-2, responsible for elimination of intracellular *S. aureus* (146–149). Perforin-2 is found suppressed in chronic diabetic wounds associated by microbial dysbiosis and persistent inflammation allowing for intracellular accumulation of *S. aureus* in epidermal keratinocytes (146). Collectively, these findings warrant further investigation into ability of HS pathogens to escape host innate immunity.

## Genetic component of HS

There are multiple genetic factors that correlate with a patient’s susceptibility to develop HS. Approximately 30% of HS patients have a positive family history (2, 5, 150, 151). A recent Dutch nationwide twin cohort study reported a 77% calculated narrow sense heritability in HS which suggests a strong genetic component in pathogenesis of the disease (152).

Mutations in genes encoding γ-secretase, a protease that targets several type-1 transmembrane proteins such as amyloid precursor proteins and the Notch protein family, are considered the most common in HS (153) although HS can be also classified as polygenic condition (151). About 36 mutations in genes encoding γ-secretase, including *NCSTN*, *PSEN1*, and PSENEN, were identified in specific patient populations with severe HS (2, 150, 154). These mutations impair γ-secretase activity impairment which may lead to alterations in keratinocytes proliferation and differentiation *via* impaired Notch signaling. Deregulated Notch signaling in HS may contribute to many of the major pathogenic events in HS. Notch signaling also plays a role in suppressing proinflammatory cytokine release *via* negative feedback inhibition of TLR4-triggered macrophages usually induced by bacterial lipopolysaccharides (155). Increased proinflammatory cytokine release contributes to activation of Th17-driven inflammation, a major immunologic characteristic of HS (156). Natural killer cells (NKs) whose development is mediated by Notch signaling are diminished in HS (157, 158). Besides Notch signaling, γ-secretase mutations may also induce dysregulation of the complement system. γ-secretase can cleave CD46, a regulatory cofactor of protease Factor I responsible for cleaving C4b and C3b deposited on cells into more benign products to protect host cells (159, 160). Binding of CD46 to C3b dimers induces production of regulatory T cell phenotypes such as T-regulatory cell 1 which then secretes large quantities of IL-10 (161, 162). CD46 expression and function is well documented to be impaired in cutaneous autoimmune disorders such as systemic lupus erythematous and bullous pemphigoid (163). Inhibited γ-secretase may allow for disruption of the protective regulatory function of CD46 and therefore inflammation *via* augmented complement and T-cell activity (127).

Several other genetic associations have been observed in HS, including *NOD2* mutations (164). In addition, RNA expression of NOD2 has been demonstrated to be increased in lesional HS skin as compared to perilesional skin. This response was positively correlated with other AMPs such as hBD2, S100A7, and RNAse 7 (117). However, genomic analysis targeting *NOD2* polymorphisms has not yet isolated any of significant correlation to HS (165). Additionally, the presence of over six copies of the *DEFB* gene cluster, was shown to hold a significantly increased risk of developing more severe HS disease (61). This is hypothesized to be a consequence of the loss of hBD-2’s protective action against bacterial superinfection of the skin, specifically against *S. aureus.* This same association is not unique for HS as it is also present in psoriasis, a disease that has well-established ties with dysregulation of AMPs including hBD-2 and hBD-3 (61). However, as the most HS patients with a positive family history (>90% in white individuals) do not have γ-secretase or other mutations (2, 5), genetic features contributing to HS development still need to be investigated.

## Treatment strategies

Considering the role of bacteria and the immune system in the pathogenesis of HS, the use of antibiotics and biologics with anti-inflammatory effect is a direct extension. Antibiotics, both topical and systemic, are often used as a first line therapy in treating HS. Systemic tetracycline therapy is amongst the most prescribed therapy along with clindamycin, rifampin, and dapsone (166). These classes of antibiotics have well documented anti-inflammatory action that, along with their antibacterial activity, is crucial in treating HS.

Biologic therapies that target specific immune pathways are an emerging class of treatment in HS. Biologics have shown efficacy in several inflammatory conditions such as inflammatory bowel disease (IBD) and psoriasis. Adalimumab, an anti-TNFα monoclonal antibody, that demonstrated efficacy in large placebo controlled trials and is currently the only FDA-approved biologic for HS treatment (167). In addition, several biologics are currently being investigated for efficacy in HS. Anakinra, an IL-1 receptor antagonist, has been demonstrated to decrease disease severity and improve quality of life in two clinical studies (168, 169). Bermekimab is a human IgG1k monoclonal antibody that also targets Il-1 that has been shown to be effective in treating HS patients that have failed anti-TNFα therapy likely due decreased neovascularization and modulation of IL-8 and hBD-2 production (170, 171). Secukinumab, a monoclonal antibody against IL-17, reported achievement of HS clinical response in 67% of patients in a pilot trial (172). A retrospective series and anecdotal data demonstrated that guselkumab, an anti-IL-23 monoclonal antibody, may be efficacious in treating HS (173).

Biologics can be used in conjunction with surgical therapy in patients with severe disease to maximize clinical response (174). Surgical procedures can be focused, such as deroofing procedures or skin-sparing excision with electrosurgical peeling, or more extensive such as wide excisions, and wound management in HS remains a large burden for patients (175). Currently, there is a paucity of HS-specific wound dressing options available. Given the interplay between the immune system and the dysbiosis that is present in HS, combination of therapies that target both of these disease elements simultaneously is likely to have a synergetic effect. However, currently such combination therapy protocols do not exist and quality data to prove the efficacy of this strategy is missing.

## Discussion

HS lesional skin is characterized by activation of both innate and adaptive immune response in conjunction with microbial dysbiosis reflected in upregulation of multiple AMPs, complement system proteins, and numerous cytokines (**Figure 1**). However, it remains to be fully understood whether the dysregulation of immune responses is causative of or consequence of microbiota dysbiosis. In HS, progressive microbial dysbiosis likely drives AMP and complement dysregulation associated with activation of the innate immune response. These features all contribute to excessive inflammation, that is ineffective in eliminating pathogens (**Figure 1**). The net result is a vicious cycle that results in local tissue destruction, disease progression and potentially, systemic elaboration of cytokines that spreads the disease in genetically, microbiologically, and immunologically susceptible individuals. Future studies should tease out the primary event and the setting required for this vicious progressive cycle to develop, clarify the directionality of this relationship, and identify molecular pathways through which this is mediated. Advancing our understanding of the interplay between microbial dysbiosis and immune dysregulation may provide direction into developing new therapeutics for treating HS.

Initial findings based on aberrant complement activation at the systematic level (132), have resulted in two ongoing clinical trials targeting the C5a–C5aR1 axis (135, 176, 177). However, the variability of results in complement levels amongst studies also suggests benefit in correlating results with patients’ genotype and/or microbiome shifts in response to therapy. In addition, the only US-FDA approved biologic therapeutic adalimumab, an anti-TNF-α monoclonal antibody, has shown limited efficacy (178). Lower efficacy of biologic drugs may in part be a consequence of persistent microbial dysbiosis, particularly bacterial biofilms of anerobic Gram-negative bacteria in HS tunnels at advanced stages of the disease. Like HS, IBD has demonstrated altered expression of AMPs, aberrant inflammatory response, and microbial dysbiosis that has been hypothesized to contribute to resistance to biologic therapy. For example, dysbiosis of bacteria that produce short chain fatty acids (SCFAs) in the gut microbiota of IBD patients has been associated with resistance to anti-TNFα therapy (179). While SCFAs inhibit biofilm formation (180, 181), diminished concentrations of SCFA-producing bacteria in biologic-resistant IBD patients suggests that resistance to therapy has microbial mediated mechanism. The findings in patients with IBD and similar immunologic features of IBD and HS (182) support the importance of prospective studies on microbiome in HS and its changes in patients undergoing biologic therapy. Future research should also aim to investigate combination of therapies targeting the deregulated immune response and microbial dysbiosis should be evaluated.

Based on deregulation of innate immune responses and initial changes in HS microbiome, antibacterial therapy may have important role in limiting progression of early HS. However, considering the rise in antibiotic resistance, antimicrobials may primarily manage disease by stalling inflammation, rather than eliminating pathogens. Additionally, the data presented in this review can reasonably predict the efficacy of a carbapenem antibiotic ertapenem in HS as well as its inability to maintain long term results (183).

Therefore, it is not surprising that the standard of care for moderate to severe disease (i.e. the presence of tunnels) is moving towards a combination of medical and surgical therapy (174). Deroofing is a common surgical procedure in which tunnels are selectively removed. In two large uncontrolled series, the ability of deroofing to prevent recurrence seems to decrease with disease progression. Milder HS was associated with 17% recurrence after deroofing (184), and more severe disease with 41% recurrence (185). The efficacy of deroofing may be related to the elimination of biofilms and reversal of the vicious cycle discussed above. Emerging clinical data suggests that topical anti-biofilm therapy that is administered without removing the diseased skin can eliminate isolated tunnels (186), and new biofilm-eliminating approaches should be considered (187, 188). Future metagenomics studies should examine the antimicrobial resistance in HS lesions and tunnels at advanced stages of the disease and characterize the interaction between the microbiome, biofilms, and innate immunity.

This review has summarized the potential contributions of innate immune system activation and microbial dysbiosis to HS pathogenesis. While studies utilizing single cell omics approaches have already revealed insight into HS pathology and deregulation of host responses (189–191) advancements in spatial transcriptomics and proteomics should be utilized to further understanding of deregulated innate immunity at the host-pathogen interphase. Future longitudinal studies simultaneously evaluating the focal genetic makeup, innate immunity, microbial dysbiosis and bacterial biofilms will provide detailed insights in HS pathogenesis and progression.

## Author contributions

IP and HL-T conceived the review and coordinated writing. DC, RA, WA, ML, NS, MT-C, HL-T, and IP were involved in literature search and writing, and had final approval of the submitted and published versions of the manuscript.

## Acknowledgments

We are thankful to all current and past members of our research teams for continuous inspiration and support. The figure in this manuscript was created using Biorender.com. This work is in part supported by Hidradenitis Suppurativa Foundation Danby Research Grant (IP and NS).

## Conflict of interest

ML has served on the advisory boards for Abbvie, InflaRx, Janssen, and Viela Bio, and consulted for Almirall, BSN medical, Incyte, Janssen, Kymera, Phoenicis, and XBiotech.

The remaining authors declare that the research was conducted in the absence of any commercial or financial relationships that could be construed as a potential conflict of interest.

## Publisher’s note

All claims expressed in this article are solely those of the authors and do not necessarily represent those of their affiliated organizations, or those of the publisher, the editors and the reviewers. Any product that may be evaluated in this article, or claim that may be made by its manufacturer, is not guaranteed or endorsed by the publisher.
